# The Importance of Liver‐Lung Communication in Pulmonary Vascular Diseases

**DOI:** 10.1002/cph4.70140

**Published:** 2026-03-31

**Authors:** Navneet Singh, Hilary M. DuBrock, Sasha Z. Prisco, Zhiyu Dai, Qi Zheng, Michael B. Fallon, Thenappan Thenappan, Corey E. Ventetuolo, Arun Jose

**Affiliations:** ^1^ Alpert Medical School of Brown University Providence Rhode Island USA; ^2^ Mayo Clinic Rochester Minnesota USA; ^3^ University of Minnesota Minneapolis Minnesota USA; ^4^ Washington University in St. Louis St. Louis Missouri USA; ^5^ University of Arizona College of Medicine‐Phoenix Phoenix Arizona USA; ^6^ University of Cincinnati Cincinnati Ohio USA

## Abstract

In normal health, the liver and lungs enjoy a close anatomic, physiologic, and functional relationship. In the context of pulmonary vascular disease, however, there is accumulating evidence that the interplay between the gut microbiome, hepatic system, and pulmonary vasculature (so‐called “gut‐liver‐lung” axis) plays an important role in driving disease pathogenesis and determining clinical outcomes. Despite recognizing the importance of the gut‐liver‐lung axis in pulmonary vascular disease however, little is known about the clinical characteristics, circulating factors, and physiologic pathways that mediate this important axis of communication. In this clinical and translationally focused review, we provide an overview of liver‐lung communication in normal physiology, and contrast this with gut‐liver‐lung derangements in pulmonary arterial hypertension, portopulmonary hypertension, and hepatopulmonary syndrome. We conclude with identifying key gaps in knowledge that will need to be addressed in order to manipulate the gut‐liver‐lung axis to prevent worsening pulmonary vascular disease, develop novel therapeutics, and improve patient outcomes.

## Graphical abstract

Schematic depicting the key drivers of liver‐lung communication in pulmonary vascular diseases, including hepatic dysfunction, portosystemic shunting, and gut microbial dysbiosis, which together drive pulmonary vascular remodeling.

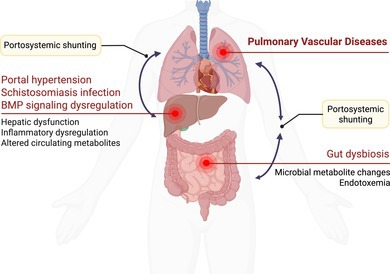

## 1 Introduction

**FIGURE 1:**
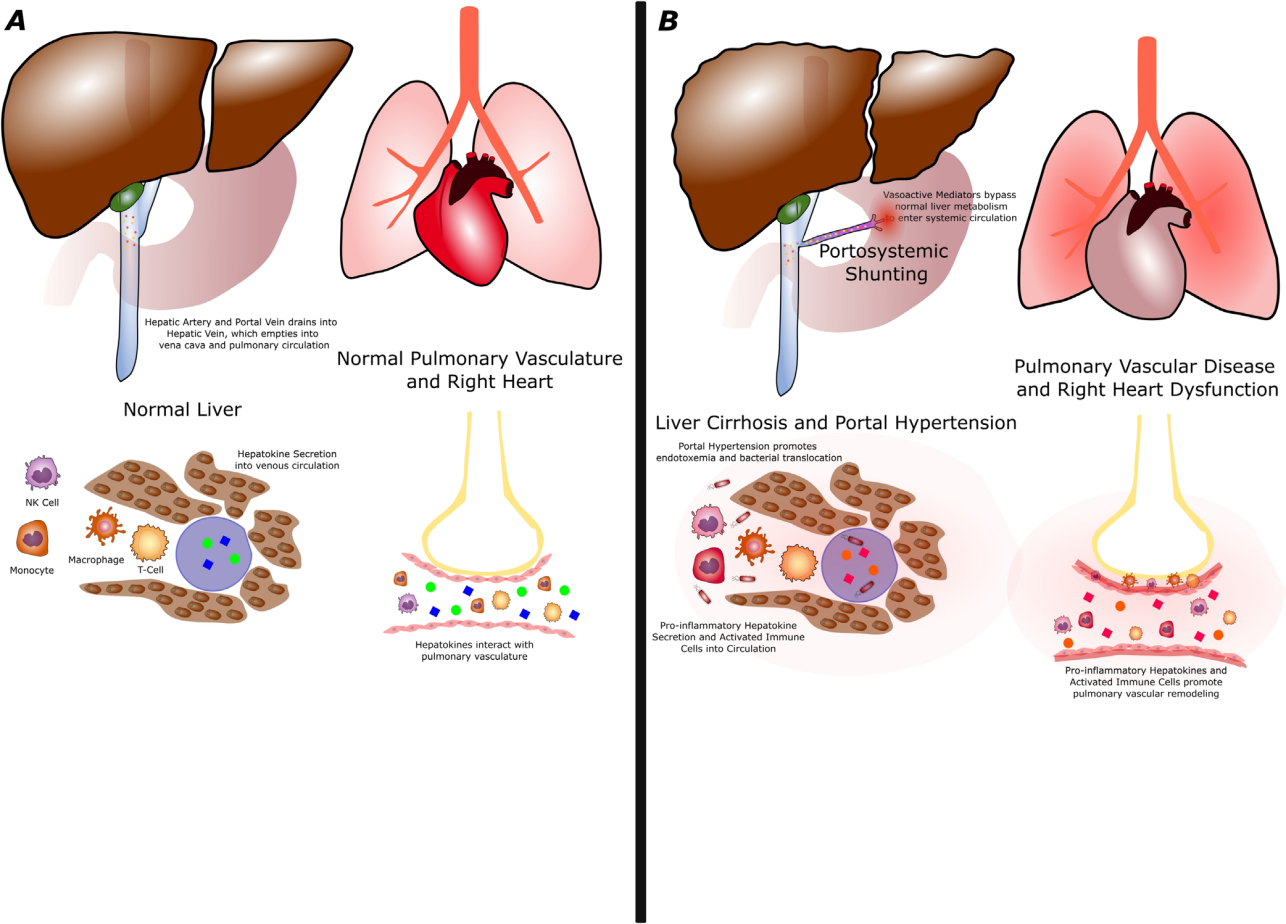
Contrast between normal liver‐lung communication in health and disease. (A) Normal liver‐lung communication, with hepatic artery and portal venous blood draining into the hepatic vein, and emptying in to the vena cava and pulmonary circulation. Appropriate hepatokine secretion by leukocytes into the venous circulation interacting with the pulmonary vasculature to maintain normal pulmonary vascular and right heart structure and function. (B) Mechanisms by which abnormal liver‐lung communication may drive pulmonary vascular disease, including portosystemic shunting releasing vasoactive mediators into systemic circulation, liver dysfunction and portal hypertension promoting endotoxemia, leukocyte activation, and pro‐inflammatory cytokine release into circulation, and pro‐inflammatory stimuli and activated immune cells ultimately promoting pulmonary vascular remodeling and right heart dysfunction.

Liver dysfunction is increasingly recognized as an important feature of pulmonary vascular disease. Not only do liver impairments influence pulmonary arterial hypertension (PAH), but there are two distinct pulmonary vascular diseases that only occur in the context of liver disease: portopulmonary hypertension (PoPH) and hepatopulmonary syndrome (HPS). While effective bi‐directional liver‐lung communication is necessary to maintain human health, disrupted cross‐organ communication can drive disease pathogenesis. Despite being recognized as a distinct disease entity over 30 years ago, fundamental questions regarding PoPH and HPS remain unanswered. The optimal role of liver transplantation (LT) in these conditions continues to evolve, the mechanisms and circulating hepatic “factors” that drive disease remain undefined, and recognition that hepatic dysfunction can affect PAH pathogenesis (and vice‐versa) is a fairly recent development. Additionally, though the intestinal gut microbiome sits in close proximity anatomically and functionally to the liver, our exploration of the importance and contributions of intestinal gut dysbiosis to pulmonary vascular disease remains in its infancy (Figure 1). In this context, and given the rapid evolution of the field in just the last decade, a concise overview of the liver‐lung axis (or gut‐liver‐lung axis) in pulmonary vascular disease is long overdue. By examining the contributions of the liver and intestinal gut microbiome to pulmonary vascular disease, with a special focus on the use and consequences of LT in PoPH and HPS, this article highlights key knowledge gaps that need to be addressed to advance our understanding of the liver‐lung axis in pulmonary vascular disease. Ultimately, we hope this review lays the groundwork to facilitate improvements in diagnosis, management, and clinical outcomes for patients with these conditions.

## 2 Liver‐Lung Communication and the Hepatopulmonary Circulation

**FIGURE 2:**
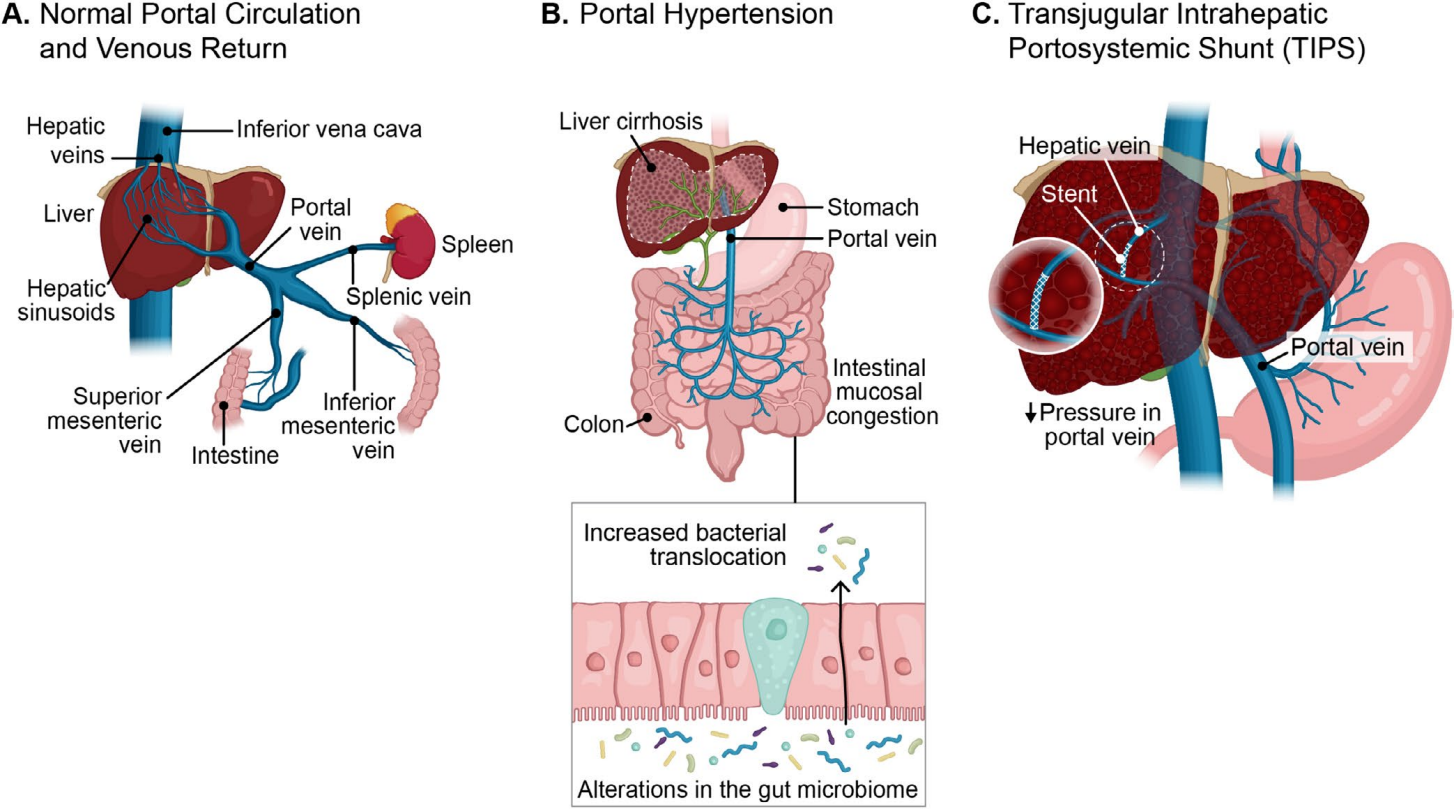
Potential contributions of the gut‐liver axis to PAH pathogenesis. (A) Normal portal venous circulation. (B) In portal hypertension, there can be increased splanchnic/intestinal venous congestion, which may alter the gut microbiome and increase bacterial and metabolite translocation. (C) When blood is shunted from the portal to hepatic vein with a TIPS procedure, the resulting bypass of normal hepatic filtration may increase the risk of developing portopulmonary hypertension. Figure designed with the assistance of Cynthia Faraday.

In normal physiology, the liver and lung enjoy a close anatomic and functional relationship (Umek et al. 2023; Lautt 2009) (Figures 1A and 2A). The liver is unique in that it receives a dual vascular blood supply, with approximately 25% of blood coming from the hepatic artery and the remaining 75% received from the portal vein. The portal vein is the predominant venous drainage for the gut, arising from the confluence of the superior mesenteric vein and the splenic vein (which drains the inferior mesenteric vein). The portal circulation is a low‐pressure circuit, and although portosystemic anastomoses exist in the esophageal, rectal, umbilical, splenic, and colonic veins, the low pressure in the portal vein normally keeps these potential shunts closed (Umek et al. 2023; Lautt 2009). This unique configuration allows the liver to participate in a myriad of functions including central roles in energy homeostasis and metabolism, hormone regulation, drug detoxification, and immune system regulation. The liver has a particularly outsized role in overseeing the immune system, as it not only contains a large reservoir of immune cells (macrophages, NK cells, and T‐cells), but also produces important protein mediators of immunity (such as complement proteins, C‐reactive protein, Hepcidin, and PCSK9) and secretes a variety of hormones, cytokines, and chemokines (so‐called “hepatokines”) that direct distal immune cell functions (macrophage activation, T‐cell activation and infiltration, neutrophil degranulation, antigen presenting cell activation, etc.) (Zhao et al. 2023). The hepatic circulation is closely linked to the pulmonary vasculature, with blood draining from the hepatic circulation to the inferior vena cava, mixing with the superior vena cava, and ultimately entering the right heart and pulmonary circulation. Thus, there is also an anatomic basis for liver‐lung communication. Functionally, this allows hepatokines and liver‐derived substances to directly influence and regulate the pulmonary vasculature, forming the basis of the liver‐lung axis.

The consequence of this close relationship between liver and lung is that, in the context of hepatic dysfunction, the pulmonary parenchyma and pulmonary vasculature are also vulnerable to injury (Figures 1B and 2B). Liver dysfunction can result in impaired clearance of bacterial pathogens and byproducts, altered hormone and endocrine signaling, and failed metabolism and detoxification of circulating cytokines, vasoactive mediators, and inflammatory molecules, all of which can precipitate or exacerbate pulmonary injury. The liver‐lung axis has been implicated in several pulmonary diseases, including pulmonary vascular disease, pneumonia and acute respiratory distress syndrome (ARDS), and chronic obstructive pulmonary disease (COPD) (Herrero et al. 2020; Guillot and Tacke 2019; Hofmann 1999; Zheng et al. 2024).

Abnormal physiology and disruption of the anatomic relationship between liver and lung (such as blood bypassing the liver in the case of portosystemic shunting) have also been implicated in pulmonary vascular disease pathogenesis. Portosystemic shunting can be acquired (most commonly in the context of cirrhotic liver disease with portal hypertension, but also in the setting of venous thromboses of the portal, splenic, hepatic, and caval veins) or congenital (Abernethy malformation, where embryonic vascular persistence results in mesenteric and splenic vein drainage directly into the vena cava, bypassing hepatic circulation) (Kwapisz et al. 2014; Silva et al. 2025; Molano et al. 2020). Regardless of the cause, however, portal hypertension and portosystemic shunting of blood uniformly result in growth of fragile variceal vessels at the sites of normally closed portosystemic anastomoses (esophagus, rectum, umbilicus, spleen, and colon), bypass of normal liver metabolism and detoxification processes, increased risk of hepatic encephalopathy and hyperammonemia, and potential development of pulmonary vascular disease.

Thus, although the close relationship between liver and lungs is crucial for normal immune function, energy homeostasis, and other important bodily functions, it is also a double‐edged sword that leaves the lungs vulnerable to secondary compromise in the setting of liver injury. In the following sections, we will explore how hepatic dysfunction has been implicated in the pathogenesis of pulmonary vascular diseases, and how a deeper knowledge of cross‐organ communication could ultimately facilitate therapeutic manipulation of the liver‐lung axis to prevent or ameliorate disease and improve outcomes in this high‐risk but poorly understood patient population.

## 3 Liver‐Lung Interactions in PAH

Liver dysfunction in PAH has traditionally been ascribed to be a long‐term consequence of pressure overload from right ventricular (RV) dysfunction leading to hepatic “congestopathy”. However, as systemic manifestations of PAH gain attention (Singh et al. 2024), observations that the liver may potentiate a feed‐forward or bi‐directional relationship with the pulmonary vasculature are challenging conventional concepts (Figure 1B).

It is recognized that intrinsic liver dysfunction can lead to PAH. Schistosomiasis‐associated PAH is a form of disease caused by hepatosplenic schistosomiasis, which in turn causes pulmonary vascular mechanical obstruction by worm eggs, inflammatory insult to the pulmonary endothelium or portal hypertension, and pulmonary overflow (Knafl et al. 2020; Graham et al. 2010; Lapa et al. 2009). In other chronic liver diseases, a hyperdynamic circulation is thought, in part, to contribute to the development of Portopulmonary Hypertension (PoPH) as discussed later (Kawut et al. 2008).

Mechanisms that explain how liver dysfunction contributes to PAH development remain understudied. In left‐heart disease, pro‐inflammatory signaling from the liver mediates coronary endothelial dysfunction (Salah et al. 2021). Similarly, hepatic ketone metabolism has been linked to activation of the NLRP3 inflammasome in a rodent model of pulmonary hypertension (Blake et al. 2023), providing a potential connection between hepatic dysfunction and the systemic inflammation that is characteristic of PAH. Bone morphogenetic proteins (BMPs) are signaling ligands that are central to the pathobiology of PAH, including BMP9 which is produced in the liver (Owen et al. 2020; Rochon et al. 2020). Intrinsic liver disease in PAH patients may interrupt BMP9's role in maintaining vascular homeostasis and thus contribute to the development of pulmonary vasculopathy (Jiang et al. 2021). Sotatercept is a first‐in‐class activin‐ligand trap that “re‐balances” BMP signaling, including BMP9 (Hoeper et al. 2023). Sotatercept treatment is associated with the development of telangiectasias and intrapulmonary vascular dilatations (IPVD) in a subgroup of PAH patients (Olsson et al. 2025; Mitchell et al. 2025), whether visceral arteriovenous malformations can occur and lead to a hepatopulmonary syndrome (HPS)‐like phenotype (BMP9's role in HPS is reviewed below) remains unknown (Mullin and Ventetuolo 2025). Reinforcing the link between inflammation, BMP signaling, and liver‐lung interactions in PAH, a recent case report of PoPH patient treated with sotatercept describes the subsequent development of HPS, alongside alterations in pulmonary artery leukocyte gene expression and plasma pro‐inflammatory protein levels (Jose et al. 2026). Dedicated studies are needed to determine the mechanisms by which impaired hepatic metabolism influences inflammatory insults and disrupts vascular homeostasis in the pulmonary circulation, so that we may more effectively target these pathways for therapeutic benefit.

While chronic liver disease can worsen PAH (e.g., PoPH, schistosomiasis‐PAH), recent observations suggest the liver may also modulate outcomes in PAH in the *absence* of clinical liver disease. In a retrospective study of individual participant data from clinical trials submitted to the FDA that included PAH patients without liver disease, serologic evidence of cholestatic liver injury was linked to a lower six‐minute walk distance and higher risk of clinical worsening, hospitalization, and death compared to those without liver injury, suggesting bile acids may play a role in PAH pathogenesis (Scott et al. 2024). Recently, deficiency of the nuclear receptor coactivator 7 (NCOA7) and subsequent impairment of lysosomal acidification has been shown to increase bile acid production and immunoactivation of the pulmonary vascular endothelium (Harvey et al. 2025). Together, this work reinforces the emerging concept that the liver influences PAH along a continuous spectrum from subclinical to overt hepatic dysfunction.

## 4 Metabolite and Gut‐Liver Derangements in Pulmonary Vascular Disease

In addition to the liver‐lung interactions in PAH, accumulating evidence supports a gut‐liver‐lung axis (Figures 1B and 2B). The gastrointestinal system contains trillions of microorganisms, known as the gut microbiome (Thenappan et al. 2019). Emerging preclinical and clinical findings point to the potential role of the microbiome in PAH and pulmonary vascular disease pathogenesis (Prisco et al. 2025). Multiple studies have shown that rodent models of pulmonary hypertension (PH) demonstrate alterations in the intestinal microbiome, commonly referred to as gut dysbiosis (Callejo et al. 2018; Hong et al. 2021; Luo et al. 2021; Cao et al. 2024; Luo et al. 2023; Chen et al. 2022; Sharma et al. 2020; Nijiati et al. 2021; Adak et al. 2014).

A key mechanism by which gut microbiota interact with their host is through metabolites, which are intermediate or end products of microbial metabolism (Krautkramer et al. 2021), derived from microbial processing of dietary or other host substrates. Early observations of pulmonary arteriovenous malformations that develop after the creation of a cavopulmonary shunt in children with congenital heart disease led to hypotheses that vasoactive mediators can escape hepatic metabolism and influence the pulmonary circulation (Duncan and Desai 2003; Freedom et al. 2004; Al‐Naamani and Roberts 2013). Supporting this hypothesis, patients with Abernethy malformation (congenital portosystemic shunts) suffer from an increased incidence of PoPH and HPS, and correction of these portosystemic shunts can help mitigate the severity of PoPH and HPS (Baiges et al. 2020; Iida et al. 2010). Additionally, those with liver cirrhosis who undergo a transjugular intrahepatic portosystemic shunt (TIPS), where blood is shunted from the portal to hepatic vein via a stent to reduce portal hypertension, may also be at risk of developing PoPH (although this link comes from case reports and low‐quality evidence) (Tatah et al. 2021) (Figure 2C).

The portal venous circulation contains high microbial metabolite concentrations, which can prime immune cell function, with these activated immune cells subsequently entering systemic circulation and potentially contributing to the development of pulmonary vascular disease (Wang and Mackay 2024). Experimental models have suggested that supplementation of the short chain fatty acid butyrate, derived from intestinal microbial fermentation, may be beneficial in mitigating the development of PH in rodent models (Karoor et al. 2021). In contrast, the microbial metabolite trimethylamine N‐oxide (TMAO) may actually worsen PH, and circulating concentrations of TMAO have been linked to coronary atherosclerosis and left heart failure (Huang et al. 2022; Tang et al. 2024; Budoff et al. 2025). More globally, modulating the microbiome as a therapeutic adjunct in PAH has shown promise. An early phase safety and feasibility study of microbiota transplant therapy (MTT) in 11 PAH participants demonstrated that MTT is safe, feasible, and leads to a transient reduction of circulating proinflammatory cytokines, as well as a trend towards improved functional capacity (six‐minute walk distance) and PAH‐specific quality of life (emPHasis‐10) (Moutsoglou et al. 2025).

In addition to microbiota transplant therapy, potential approaches to modulate the gut microbiome in pulmonary vascular disease include dietary interventions and fasting, prebiotics, probiotics, postbiotics (inanimate microorganisms or their components), and antibiotics, to name a few. These approaches are reviewed in greater detail elsewhere (Prisco et al. 2025). Regarding manipulation of the gut‐liver‐lung axis for therapeutic benefit in pulmonary vascular disease patients, evidence is scant. The antibiotic norfloxacin did not reduce the portal venous pressure gradient (Kemp et al. 2009) in patients with portal hypertension, nor improve gas exchange (Gupta et al. 2010) in patients with HPS. Dietary supplementation of the amino acid carnitine, while well tolerated, did not appreciably change measures of right ventricular dysfunction or functional capacity in PAH patients (Brittain et al. 2024). In contrast, dietary modification with nutritional ketosis successfully reversed metabolic syndrome and secondary pulmonary hypertension in a case report (Kim et al. 2021). Though select examples, these data underscore both the potential and perils in effective therapeutic manipulation of the gut microbiome in mitigating pulmonary vascular disease, a field that currently remains largely unexplored.

Although gut dysbiosis has been associated with PAH, no studies to date have specifically examined the role of the gut microbiome in PoPH or HPS. This is despite the interconnected relationship between the liver and intestines, with blood from the small and large intestine draining into the mesenteric veins, converging with splenic circulation to form the portal vein as the main delivery route for blood to the liver. Resident macrophages in liver sinusoids (Kupffer cells) are responsible for phagocytosis of bacteria and endotoxins in hepatic blood (Basit et al. 2025), which then enters the inferior vena cava, the right side of the heart, and pulmonary circulation. In disease states, liver cirrhosis and portal hypertension can cause intestinal mucosal congestion, increase intestinal permeability, disrupt gut microbiome composition by reducing species diversity and richness, elevate systemic inflammation, and bypass normal bacterial and endotoxin filtering via portosystemic shunting, all of which may contribute to pulmonary vascular remodeling (Qin et al. 2014; Hao et al. 2024; Fukui 2019; Gulyaeva et al. 2025). Animal models have shed insight into possible mechanisms driving gut‐liver‐lung communication in pulmonary vascular disease. In a rat model of HPS induced by common bile duct ligation, elevated circulating endotoxin levels led to activation of pulmonary intravascular macrophages and subsequent vascular remodeling (Thenappan et al. 2010). In a separate study, treatment of such rats with the antibiotic norfloxacin decreased gram‐negative bacterial translocation via portosystemic shunting, presumably reducing pulmonary delivery of gut‐derived endotoxin, and resulting in both decreased density of pulmonary macrophages and reduced severity of HPS (Rabiller et al. 2002). Unfortunately, a subsequent human study of antibiotic therapy failed to ameliorate gas exchange abnormalities in HPS subjects, highlighting the challenges in translating mechanistic insights of liver‐lung communication into pulmonary vascular disease therapeutics (Gupta et al. 2010).

Thus, although some links have been established, the exact contribution of gut microbial dysbiosis and associated gut‐derived metabolites to portosystemic shunting and pulmonary vascular development, and how this translates to treatment of PAH, PoPH, and HPS, requires further investigation.

## 5 Mechanisms and Clinical Consequences of HPS and LT

HPS, a complication of chronic liver disease and portal hypertension, is estimated to afflict 4%–32% of LT candidates. It is characterized by intrapulmonary vascular dilations (IPVDs) and arterial hypoxemia (defined by reduced arterial oxygen tension, PaO_2_, below 80 mmHg or an elevated alveolar‐arterial oxygen gradient), disrupting ventilation–perfusion matching and gas exchange (Fallon et al. 2008; Zaka et al. 2025). Clinically, HPS impairs exercise tolerance and quality of life, worsens survival, and influences both transplant eligibility and organ allocation prioritization (Pinto et al. 2021).

HPS presents with a wide spectrum of severity, ranging from asymptomatic hypoxemia to more pronounced symptomatology including exertional dyspnea, cyanosis, and digital clubbing. Data from large epidemiologic studies have indicated HPS patients suffer from a greater burden of portal hypertension complications, reduced functional capacity, a distinct circulating peptide angiogenic signature, and increased mortality irrespective of the severity of hypoxemia (Kawut et al. 2022). Diagnosis primarily relies on contrast‐enhanced transthoracic echocardiography (i.e., the “bubble test”) to detect IPVD, frequently supplemented by 99mTc‐macroaggregated albumin scanning to quantify shunt fraction. HPS is associated with significantly reduced survival both before and after LT, which remains the only definitive treatment. Most recipients experience marked improvement in arterial oxygenation within weeks to months post‐LT, resulting in long‐term survival comparable to that of transplant patients without HPS, even in patients with severe hypoxemia before LT (e.g., PaO_2_ < 50 mmHg) (Pinto et al. 2021; Jose et al. 2021). Organ allocation for transplantation is prioritized using a numeric scale, the Model for End‐Stage Liver Disease (MELD) score, with higher scores increasing the likelihood of being offered transplantation. In recognition of the survival benefit afforded by LT, standardized MELD score increases for HPS have improved transplant access and outcomes for severely hypoxemic patients without compromising equitable organ allocation.

**FIGURE 3:**
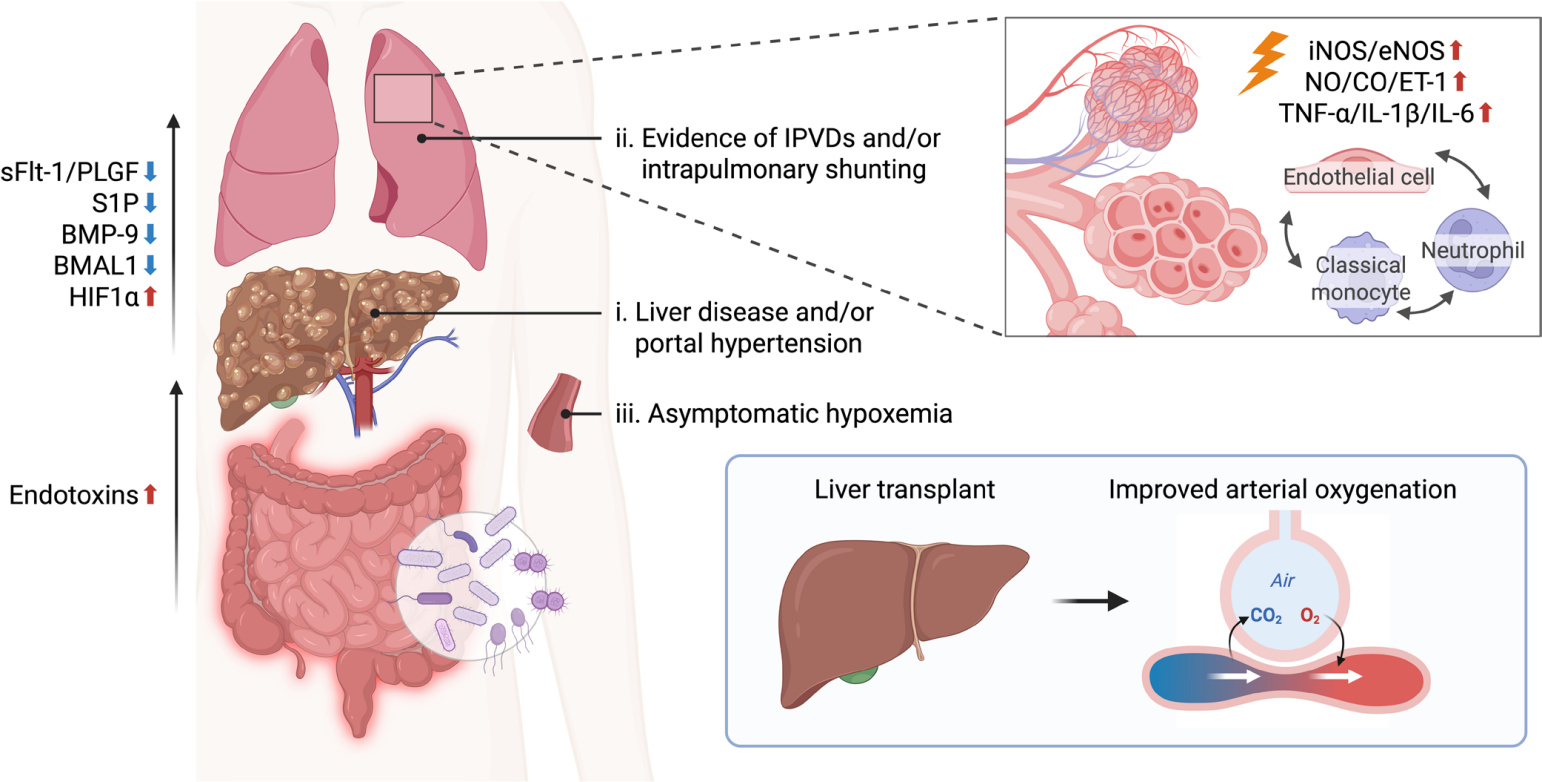
Mechanisms and clinical consequences of Hepatopulmonary Syndrome (HPS). Hepatopulmonary syndrome develops in the setting of chronic liver disease and portal hypertension and is felt to be driven by dysregulated signaling along the gut‐liver‐lung axis. Portal hypertension facilitates gut‐derived endotoxemia, leading to pulmonary recruitment and activation of macrophages, monocytes, and neutrophils, with increased expression of pro‐inflammatory cytokines including TNF‐α, IL‐1β, and IL‐6. In the lung, these inflammatory signals promote endothelial dysfunction, pathological angiogenesis, and induction of inducible and endothelial nitric oxide synthase (iNOS/eNOS), which can result in excessive nitric oxide production and intrapulmonary vascular dilatation and shunting. Several circulating mediators may contribute to disease progression, including increased placental growth factor (PlGF) activity, altered sFlt‐1/PlGF balance, reduced liver‐derived BMP‐9, depletion of sphingosine‐1‐phosphate (S1P), and disruption of hepatic BMAL1‐HIF1α signaling. Clinically, HPS is characterized by progressive hypoxemia and impaired gas exchange. Liver transplantation remains the only definitive therapy and is associated with reversal of pulmonary vascular abnormalities and improved long‐term survival.

At its core, HPS is believed to arise from a complex interplay along the gut‐liver‐lung axis, involving endothelial dysfunction, pathological angiogenesis, and excessive nitric oxide (NO) production (Figure 3). Portal hypertension promotes the translocation of gut‐derived endotoxins, activating pulmonary macrophages and monocytes, and triggering overexpression of inducible and endothelial nitric oxide synthase (iNOS/eNOS). This results in vasodilation mediated by NO, carbon monoxide, and endothelins; increasing microvascular permeability and intrapulmonary shunting (Raevens and Fallon 2018; Li et al. 2022). Proinflammatory cytokines, including TNF‐α, IL‐1β, and IL‐6, are thought to amplify monocyte recruitment to the lungs, sustaining and potentiating the inflammatory and angiogenic milieu responsible for IPVD maintenance (Raevens and Fallon 2018; Luo and Du 2022). Recent advances have identified placental growth factor (PlGF) as a stimulator of eNOS and a key mediator of HPS (Robert et al. 2025). Experimental data from both human subjects and animal models demonstrated elevated PIGF in HPS relative to non‐HPS cirrhosis patients, that PlGF promotes eNOS activation and NO overproduction in human pulmonary microvascular endothelial cells, and that PlGF deficiency or suppression reduces disease severity in rodent models of HPS. Similar translational human and animal studies have implicated the soluble vascular endothelial growth factor receptor type 1 (sFlt‐1), and the ratio of sFlt‐1 to PlGF, as candidate biomarkers specific for HPS with diagnostic potential (Raevens et al. 2015; Li et al. 2023). Sphingosine‐1‐phosphate (S1P) may also play a role, with HPS patients exhibiting depleted circulating levels of S1P, which also correlates with systemic inflammation and NO excess. Animal models also suggest activating S1P receptors mitigates pulmonary vascular injury, reduces portal pressure, and improves survival (Baweja et al. 2023).

Another critical pathway implicated in HPS involves BMP9, a liver‐derived factor essential for pulmonary vascular stability (Robert et al. 2024). As mentioned above, circulating levels of BMP9 are diminished in patients with pulmonary vascular disease (including HPS) relative to both normal patients and those with compensated cirrhosis (Owen et al. 2020). Additionally, the reduction of hepatic BMP9 expression in animal models of portal hypertension, or the knockout of BMP9 in rat models, both result in animal models manifesting the classic HPS features of IPVD and pulmonary vascular pathology. Pharmacological activation of the BMP signaling cascade, using the calcineurin inhibitor FK506, reverses IPVD and improves oxygenation in HPS animal models (Robert et al. 2024). Taken together, these studies provide strong evidence supporting a critical role of BMP9 in the pathogenesis of HPS.

Adding a novel dimension, circadian regulation also influences HPS pathogenesis (Dandavate et al. 2024). Hepatic brain and muscle Arnt‐like protein‐1 (BMAL1) and hypoxia‐inducible factor 1α (HIF1α) coordinate the hypoxic transcriptional response and link it to the circadian cycle. Curiously, mice deficient in both factors develop pulmonary vasodilation and hypoxemia through excess eNOS and NO activation, suggesting a time‐dependent regulatory mechanism may contribute to disease pathogenesis and variability.

## 6 Mechanisms and Clinical Consequences of PoPH and LT

PoPH is characterized by precapillary pulmonary hypertension that develops in the context of portal hypertension without an alternative cause. According to the 7th World Symposium on Pulmonary Hypertension, PoPH is classified as Group 1, or PAH (Kovacs et al. 2024). PoPH can develop in the setting of cirrhotic or non‐cirrhotic portal hypertension, is not strongly associated with liver disease severity, and can occur across the spectrum of liver disease etiologies (though may be more common among patients with autoimmune liver disease) (Kawut et al. 2008). PoPH is also associated with spontaneous portosystemic shunts and can develop among patients with congenital extrahepatic portosystemic shunts, even in the absence of intrinsic liver disease (Talwalkar et al. 2011).

As alluded to above, pathophysiology and disease mechanisms remain poorly defined, but portosystemic shunting of a “hepatic factor” is believed to contribute to disease pathogenesis. BMP9, a circulating vascular quiescence factor implicated in HPS, is also reduced among patients with PoPH compared to liver disease controls (Rochon et al. 2020), and augmentation of the BMP9 pathway in animal models can mitigate development of PH (Nikolic et al. 2019). However, as noted earlier, the link between BMP9 and HPS is much stronger (Robert et al. 2024), and diminished BMP9 in PoPH may be nonspecific, instead reflecting overlap with HPS and/or underlying liver dysfunction. Estradiol and estrogen metabolites are also altered among patients with PoPH, and genetic mutations in the estradiol pathway have been implicated in disease pathogenesis (Roberts et al. 2009; Al‐Naamani et al. 2021). Inflammation may also play a role. Macrophage migration inhibitory factor, a pro‐inflammatory cytokine, is elevated among patients with PoPH compared to controls with liver disease, and is associated with worse hemodynamics and survival (DuBrock et al. 2016).

PoPH is associated with symptoms of exertional dyspnea and decreased exercise tolerance, and can lead to right heart failure and death. In the absence of PAH targeted therapy or LT, overall survival is poor (Swanson et al. 2008). Compared to idiopathic PAH, patients with PoPH have worse survival despite better hemodynamics (Krowka et al. 2012). Numerous studies have shown that both liver disease severity and PH severity impact survival (DuBrock et al. 2017). Similar to idiopathic PAH, PoPH is typically treated with PAH therapy despite limited evidence since patients with PoPH have been excluded from most randomized controlled trials of PAH therapy. Meta‐analyses as well as other long‐term studies have identified that outcomes are best among patients treated with a combination of PAH therapy and LT (Deroo et al. 2020; Savale et al. 2020). Of special note, PoPH patients were excluded from all studies of the newest PAH therapeutic, the activin signaling inhibitor Sotatercept (Humbert et al. 2021; Hoeper et al. 2023; Humbert et al. 2025), so the efficacy and safety of this therapeutic is not yet established in the PoPH population. That being said, a recent case‐report suggested that while Sotatercept therapy in PoPH improves pulmonary pressures and vascular resistance, it was also associated with the concerning development of hypoxemia and HPS (Jose et al. 2026), signaling a complex and nuanced risk–benefit profile of this class of PAH therapeutics in PoPH.

A diagnosis of PoPH has significant implications for LT. Patients with severe or untreated PoPH have an increased risk of death and should not undergo LT (Krowka et al. 2000). Contraindications to LT include a mean pulmonary arterial pressure (mPAP) > 45–50 mmHg, pulmonary vascular resistance (PVR) > 5 WU or moderate to severe right ventricular dysfunction (DuBrock et al. 2025). In recognition of the improved survival for PoPH patients treated with a combination of PAH therapy and LT, patients with treated PoPH and preserved right ventricular function who meet certain hemodynamic criteria are eligible for a Model for End Stage Liver Disease exception in order to prioritize organ allocation and expedite transplantation. However, in contrast to HPS, post‐LT outcomes in PoPH are variable. In the initial post‐transplant period, PVR may increase, particularly within the initial 6 months following transplant (Savale et al. 2017). In light of this variability, current guidelines recommend continuation of PAH therapy post‐LT, with close monitoring of transthoracic echocardiogram and/or right heart catheterization to guide post‐transplant treatment decisions (DuBrock et al. 2025). Approximately half of patients are able to wean off PAH therapy post‐LT, and may experience long‐term remission from their disease (Savale et al. 2017; Cartin‐Ceba et al. 2021; Sadd et al. 2021). This is atypical for other subgroups of PAH, which are typically lifelong, progressive conditions. The remaining half of patients continue to require PAH therapy, but often experience improvement and are able to wean their medications (Savale et al. 2017). Consequently, although LT is generally not considered a “cure” for PoPH given these unpredictable outcomes, it can (and does) lead to resolution of PH in some patients. Improved understanding of the pathophysiology of PH and prediction of post‐transplant outcomes is needed to help guide management of PoPH and evaluation for LT.

## 7 Conclusions, Gaps in Knowledge, Future Directions

As succinctly summarized in the preceding sections, there is a close and interconnected anatomic, physiologic, and functional relationship between the liver, lungs, pulmonary vasculature, and intestine. Normally, this system plays key roles in immune system regulation, nutrient absorption and metabolism, bacterial and endotoxin removal, and the overall maintenance of a state of health. However, when perturbed, gut dysbiosis, hepatic dysfunction, and portal hypertension can all contribute to pulmonary vascular disease, manifesting in PAH, PoPH, or HPS.

Despite recognition that the gut‐liver‐lung axis plays a prominent role in pulmonary vascular disease, clear characterization of this inter‐organ communication pathway remains frustratingly elusive. Various hepatokines, circulating mediators, and pathways have been implicated in pulmonary vascular disease pathogenesis (bacterial endotoxemia, excess NO production, deficient BMP9, stimulated inflammation, dysregulated angiogenesis, gut‐derived circulating metabolites, etc.), but fundamental questions regarding how these intermediaries link the gut microbiome and hepatic system to pulmonary vascular remodeling remain unanswered:

- What is the role of portosystemic shunting in promoting pulmonary vascular disease due to liver disease?
- Does existence of the gut‐liver‐lung axis imply a different approach to treatment of PoPH with PAH targeted therapy to optimize clinical outcomes, or support manipulation of the gut microbiome (such as with antibiotics or probiotics) for benefit in PAH or HPS?
- Should we consider the gut‐liver‐lung axis and hepatic function when determining the optimal treatment of PAH?
- Are there liver‐lung‐specific candidate biomarkers that can serve as clinical tools to help optimize screening, diagnosis, and prognostication, or inform management decisions and guide transplantation strategies?
- Can a clearer understanding of the gut‐liver‐lung axis identify novel therapeutic targets and pathways for further development in the clinical realm?

Pulmonary vascular disease has an unacceptably high morbidity and mortality, and LT is a scarce resource. A more detailed characterization of the gut‐liver‐lung axis in pulmonary vascular disease is essential, and translational work directed at answering important questions regarding mechanisms, mediators, and clinical implications is urgently needed to advance knowledge in the field, expand our therapeutic options, and improve clinical outcomes for these patients.

## Author Contributions

All authors were responsible for the study design, wrote the initial draft, and edited and prepared the final submitted manuscript.

## Funding

This study is supported by NIH grant T32 HL134625 (Corey E. Ventetuolo, Navneet Singh); R01 HL141268 (Corey E. Ventetuolo); R01 HL174007 (Corey E. Ventetuolo); K08 HL168166 (SZP); American Heart Association Career Development Award 23CDA1049093 (Sasha Z. Prisco); R01 HL158596 (Zhiyu Dai); R01 HL162794 (Zhiyu Dai); R01 HL170096 (Zhiyu Dai); R01 HL169509 (Michael B. Fallon and Zhiyu Dai); K23 HL16497 (Arun Jose).

## Disclosure

Hilary M. DuBrock has received consulting fees or served on advisory boards for Merck, Janssen, Liquidia, Gossamer Bio, and United Therapeutics. Sasha Z. Prisco is on the speakers bureau for Merck. Thenappan Thenappan has received consulting fees or served on advisory boards for United Therapeutics, Janssen, Merck, Aria CV, Gossamer Bio, and Altavant Science. Corey E. Ventetuolo has received personal fees from Merck, Janssen, Pulmovant, and Regeneron, outside of the submitted work. Her institution has received fees for the conduct of clinical trials from Merck, Pulmovant, Tenax, 35 Pharma, Goassamer Bio, and United Therapeutics. Arun Jose has received consulting fees or served on advisory boards for Janssen, Merck, and Gossamer Bio, and has received research funding from United Therapeutics.

## Conflicts of Interest

The authors declare no conflicts of interest.

## Data Availability

Data sharing not applicable to this article as no datasets were generated or analysed during the current study.
